# A rare cervical dystonia mimic in adults: congenital muscular torticollis (fibromatosis colli)

**DOI:** 10.3389/fneur.2013.00006

**Published:** 2013-02-12

**Authors:** Hector Gonzalez-Usigli, Alberto J. Espay

**Affiliations:** Department of Neurology, Gardner Family Center for Parkinson's Disease and Movement Disorders, University of Cincinnati School of MedicineCincinnati, OH, USA

Congenital muscular torticollis (CMT), also known as fibromatosis colli, is recognized as unilateral contracture and shortening of the sternocleidomastoid (SCM) muscle due to muscle atrophy and interstitial fibrosis, causing ipsilateral head tilt and turn (Do, 2006). Its frequency ranges from 0.3 to 2% in newborns with history of perinatal injury, but it is far less common in adults, who are often misdiagnosed as cervical dystonia (Patwardhan et al., 2011). In cases recognized during adulthood, subtle torticollis may have been overlooked since infancy or become apparent in the second decade of life or later (Brans et al., 1996). We report two cases recognized in adulthood initially misdiagnosed as cervical dystonia, whereby standard antidystonic treatments were unrewarding but SCM release proved beneficial.

## Case reports

### Case 1

**Figure 1:**
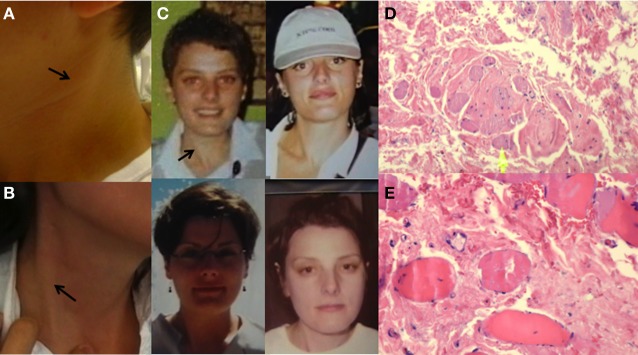
**Sternocleidomastoid appearance in Case 1 (A) and Case 2 (B).** The taut sternocleidomastoid muscle is also appreciated in older photos taken of Case 2 at the age of 20, 26, 29, and 30 years, clockwise **(C)**. H&E stained slides of muscle of Case 1 showed dense, fibrotic replacement, with collagen deposition containing scattered fibroblast nuclei and entrapped, atrophic skeletal muscle fibers at low **(D)** and high magnification **(E)**. No inflammation was seen.

This 31-year-old woman exhibited a fixed left head tilt and turn with minor limitation of cervical range of motion since childhood. Her forceps-assisted birth by vaginal delivery was not associated with perinatal injuries. During childhood, she reported persistent head tilt to the left. She had headaches and back pain presumably from compensatory efforts at straightening her neck posture. There was no sensory trick or abnormal posturing of other body segments. Palpation of her neck musculature revealed a non-tender, taut fiber within the left SCM. She had limitation of right head tilt (see Video, Segment 1, Supplementary material; Figure 1A).

### Case 2

This 37-year-old woman became aware of right head turning and bilateral shoulder pain during her initial pregnancy, 9 years previously. A few years and two pregnancies later, she noted right head tilt and mild right turn with ipsilateral shoulder pain. Prominent tightening of the right SCM became apparent. There was no sensory trick or abnormal posturing of other body segments. Palpation of her neck musculature revealed a non-tender, taut fiber within the right SCM muscle (see Video, Segment 2, Supplementary material; Figures 1B,C).

Both patients worsened with botulinum toxin type A injections (onabotulinumtoxinA, BoNT-A increased over 3–4 sessions, respectively, to 150 units) into the affected SCM but experienced substantial relief of their torticollis and associated pain following partial myectomy of the anterior belly of the SCM muscle. Biopsy in both cases demonstrated fibrous transformation of skeletal muscle fibers (Figures 1D,E), pathognomonic of CMT.

## Discussion

CMT is a rare cause of pseudodystonia expressed as torticollis, whose recognition is important in order to avoid ineffective and potentially harmful antidystonic treatments. CMT is usually recognized in neonates as a circumscribed palpable mass confined to the SCM, affected unilaterally, which may gradually disappear between 4 and 8 months of age or be associated with other orthopedic abnormalities such as hip dysplasia or lower extremity abnormalities (Morrison and MacEwen, 1982). Its recognition in the second decade or later is unusual but possible when contracture and shortening of the SCM is mild and the head tilt toward the affected side is subtle. In addition to such tilt, however, several findings should increase the diagnostic suspicion for this disorder, including the presence of a cord-like, fibrotic SCM muscle (as illustrated in the video of Case 2), the lack of a tremor component, and the absence of sensory tricks. CMT should be considered in the differential diagnosis of non-dystonic torticollis in young adults when the cervical posture is relatively fixed and a taut cord along the axis of the SCM can be palpated.

Although isolated successes have been reported with chemodenervation (Bouchard et al., 2010) the fibrotic replacement within the SCM makes BTX injections unlikely to provide sustained benefits (Collins and Jankovic, 2006). Surgical release, as shown by our cases, can be beneficial and justifies the clinical distinction of this disorder from cervical dystonia (Jones et al., 2012).

Besides CMT, other causes of pseudodystonic torticollis include focal myopathies, rotational atlantoaxial subluxation, posterior fossa tumors, congenital Klippel–Feil anomaly, syringomyelia, trochlear nerve palsy, and vestibular torticollis.
